# Supplementary material for: Synthesis and Hydrogelation of Star-Shaped Graft Copolypetides with Asymmetric Topology
Source: Gels. 2022 Jun 9;8(6):366. doi: 10.3390/gels8060366 (PMC9223145; doi:10.3390/gels8060366)
Supplement: Supplementary file 1 [file gels-08-00366-s001.zip › gels-1762761-supplementary.pdf]

gels

MDPI

Article

Synthesis and Hydrogelation of Star-Shaped Graft Copolypeptides with Asymmetric Topology

Thi Ha My Phan [1,†], Yu-Hsun Yang [1,†], Yi-Jen Tsai [1], Fang-Yu Chung [1], Tooru Ooya [2,3], Shiho Kawasaki [2] and Jeng-Shiung Jan [1,4*]

[1] Department of Chemical Engineering, National Cheng Kung University, Tainan 70101, Taiwan; n36087113@gs.ncku.edu.tw (T.H.M.P.); e34074093@gs.ncku.edu.tw (Y.-H.Y.); n36094704@gs.ncku.edu.tw (Y.-J.T.); f54081036@gs.ncku.edu.tw (F.-Y.C.)
[2] Graduate School of Engineering, Kobe University, Kobe 657-8501, Japan; ooya@tiger.kobe-u.ac.jp (T.O.); shihoshi@stu.kobe-u.ac.jp (S.K.)
[3] Center of Advanced Medical Engineering Research & Development (CAMED), Kobe University, Kobe 657-8501, Japan
[4] Hierarchical Green-Energy Materials (Hi-GEM) Research Center, National Cheng Kung University, Tainan 70101, Taiwan
* Correspondence: jsjan@mail.ncku.edu.tw
† These authors contributed equally to this work

Table S1. Characterization of star-shaped poly (Z-L-lysine) homopolypeptides (s-PZLL). The degree of polymerizations (DP), number-averaged molecular weights (M_n), and ratio of weight-averaged molecular weight to number-averaged molecular weight (M_w/M_n) were calculated from proton nuclear magnetic resonance (^{1}H NMR) and gel permeation chromatography-light scattering (GPC-LS) analyses.

Polypeptides	Feed ratio	^{1}H NMR		GPC		
		DP	M_n (g mol^{-1})	M_n (g mol^{-1})	M_w/M_n	DP
**6s- PZLL$_{32}$**	1:90	32.1	50654	50800	1.16	32.0
12s- PZLL$_{12}$	1:180	11.9	34121	32200	1.99	10.0
24s- PZLL$_{12}$	1:480	12.3	72109	55600	1.52	8.6

Table S2. Radius of gyration (R_g) of nano-assemblies in star-shaped graft copolypeptides. The concentration of all samples was 8.0 wt%. The data was fitted from Small-angle X-ray scattering (SAXS) analysis using SasView software (version 5.0.4).

Polypeptide	R_g(Å)
12s-PLL$_{12}$-*g*-Indo$_{0.10}$	151
12s-PLL$_{12}$-*g*-Phenyl$_{0.08}$	161
24s-PLL$_{12}$-*g*-Indo$_{0.11}$	215
24s-PLL$_{12}$-*g*-Phenyl$_{0.13}$	192

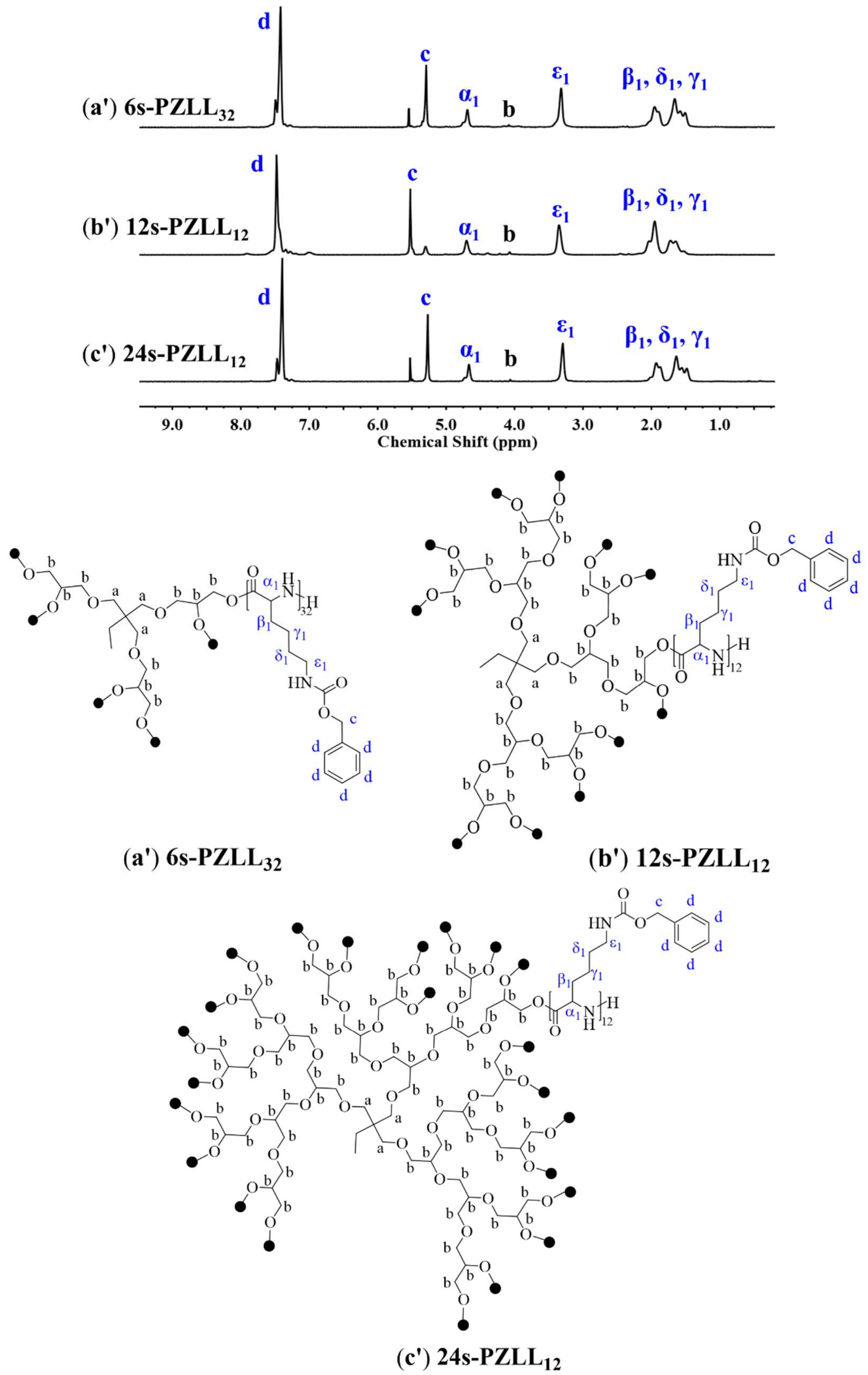

Figure S1. ^{1}H NMR of (**a′**) 6s-PZLL$_{32}$, (**b′**) 12s-PZLL$_{12}$, and (**c′**) 24s-PZLL$_{12}$ homopolypeptides in trifluoroacetic acid-*d*$_1$ (TFA-*d*$_1$). The symbols were used to represent the different protons.

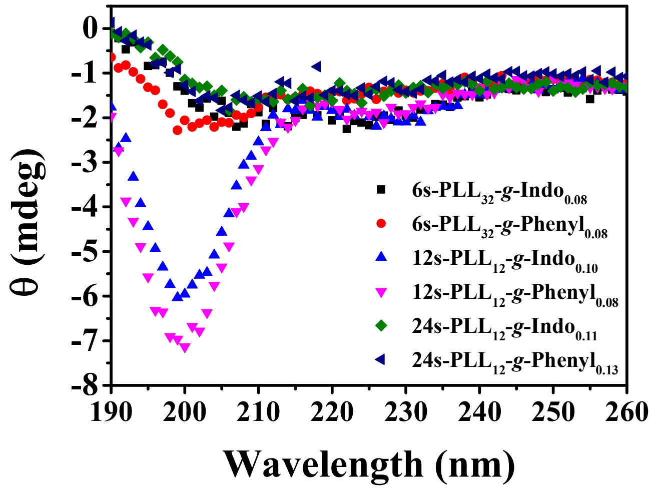

Figure S2. Circular dichroism (CD) spectra of graft polypeptides at 0.1 mg/mL.

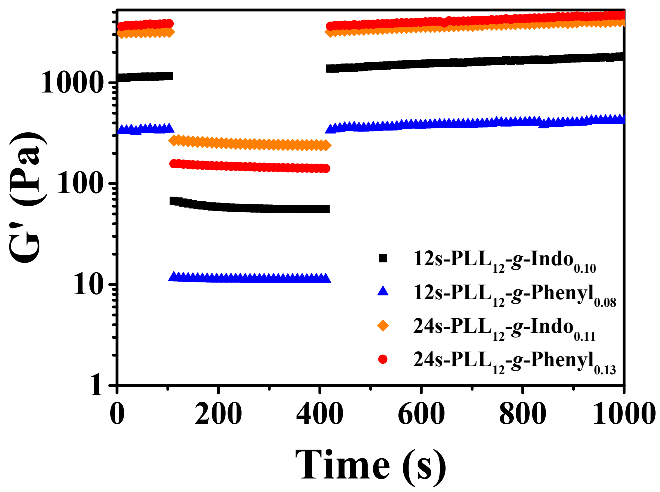

Figure S3. Recovery behavior of 12-amed and 24-armed polypeptide hydrogels. The concentration of hydrogel samples was 8.0 wt%. The rheological measurement was operated at 1 rad/s of frequency, room temperature and various strain at three continuous periods: 1.0% for 100s, 100.0% for 300s, and 1.0% for 600s.
